# End-Tidal Carbon Dioxide Monitoring for Spontaneous Pneumothorax

**DOI:** 10.1155/2021/9976543

**Published:** 2021-06-14

**Authors:** Gyeong Min Lee, Yong Won Kim, Sanghun Lee, Han Ho Do, Jun Seok Seo, Jeong Hun Lee

**Affiliations:** ^1^Department of Emergency Medicine, Dongguk University Ilsan Hospital, Dongguk University College of Medicine, Goyang, Republic of Korea; ^2^Department of Emergency Medicine, Kangwon National University College of Medicine, Chuncheon, Republic of Korea

## Abstract

**Background:**

Spontaneous pneumothorax should be classified as primary spontaneous pneumothorax (PSP) or secondary spontaneous pneumothorax (SSP) because treatment strategies may differ depending on underlying lung conditions and clinical course. The pulmonary dysfunction can lead to changes in end-tidal carbon dioxide (ETCO_2_). The aim of this study was to investigate the difference in ETCO_2_ between PSP and SSP.

**Methods:**

This retrospective observational study included adult patients diagnosed with spontaneous pneumothorax in the emergency room from April 2019 to September 2020. We divided patients into PSP and SSP groups and compared ETCO_2_ variables between the two groups.

**Results:**

There were 33 (66%) patients in the PSP group and 17 (34%) patients in the SSP group. Initial ETCO_2_ was lower in the SSP group than in the PSP group (30 (23–33) vs. 35 (33–38) mmHg, *p*=0.002). Multivariate analysis revealed that respiratory gas associated with SSP was initial ETCO_2_ (OR: 0.824; 95% CI: 0.697–0.974, *p*=0.023). The optimal cutoff for initial ETCO_2_ to detection of SSP was 32 mmHg (area under curve, 0.754), with 76.5% sensitivity and 72.7% specificity.

**Conclusion:**

ETCO_2_ monitoring is a reliable noninvasive indicator of differentiating between PSP and SSP. Initial ETCO_2_ lower than 32 mmHg is a predictor of SSP.

## 1. Introduction

Spontaneous pneumothorax (SP) without external factor is traditionally classified as primary spontaneous pneumothorax (PSP) or secondary spontaneous pneumothorax (SSP) based on the absence or presence of associated underlying lung conditions. In clinical course of SSP, SSP is usually more unstable than PSP. Thus, SPP needs longer hospitalization period, requires more surgical intervention, and leads to higher mortality. [1] Regarding management strategies, not only characteristics of pneumothorax itself, but also the underlying lung disease associated with SPP should be considered. [2, 3] Therefore, it is important to distinguish between PSP and SSP for a timely diagnosis to guide appropriate management.

Partial pressure of end-tidal carbon dioxide (ETCO_2_) can be measured via capnography. ETCO_2_ directly reflects emission of carbon dioxide (CO_2_) by ventilation and indirectly reflects the gas exchange capacity of lung and transport of CO_2_ via pulmonary circulation. Under normal physiologic conditions, the ETCO_2_ level is 35–40 mmHg. ETCO_2_ is correlated with partial pressure of carbon dioxide in arterial blood (PaCO_2_). The gradient between PaCO_2_ and ETCO_2_ (Pa-ETCO_2_ gradient) should be maintained at 2–5 mmHg [4]. However, in respiratory dead space and those with a low cardiac output that can present as ventilation-perfusion (V/Q) mismatch, Pa-ETCO_2_ gradient may be increased due to a reduction of ETCO_2_ [5–7]. Pneumothorax is one of the diseases with alveolar hypoventilation from the reduction of vital capacity by air in the pleural space. Therefore, we hypothesized that ETCO_2_ could be decreased by SP and that such decrease could be large, especially in SSP with lung dysfunction. The objective of this study was to determine whether ETCO_2_ could be used to distinguish between PSP and SSP.

## 2. Subjects and Methods

### 2.1. Study Design and Hospital Setting

This was a retrospective observational study conducted in consecutive patients aged greater than 16 years who had spontaneous pneumothorax and who had been admitted to a tertiary hospital emergency room (ER) between April 2019 and September 2020. Patients were excluded if they were not monitored for ETCO_2_ or did not perform a chest computed tomography (CT) scan.

During the study period, patients who had symptoms with dyspnea and/or suggested pneumothorax such as pleurisy (pain while breathing, shortness of breath, and cough) were admitted. We usually monitored side-stream ETCO_2_ using Cap 35 Capnography (Medtronic Inc., USA) as a portable respiratory monitor. The average value of ETCO_2_ in 5 cycles of breathing was recorded 1 minute after starting ETCO_2_ monitoring and then recorded on the electronic medical chart at intervals of about one hour. The presence of pneumothorax was confirmed through simple chest *X*-ray. A chest CT scan was routinely performed to identify underlying pulmonary diseases that could contribute to the disease course unless patient refused it. Closed tube thoracotomy was indicated clinically for unstable pneumothorax cases (such as large-size pneumothorax, bilateral pneumothorax, with hemodynamic compromise or respiratory distress). Tube management was done by emergency physicians or thoracic surgeons. Chest CT was read by a radiologist.

### 2.2. Study Variables and Definition

Clinical data were obtained from electronic medical records, including age, gender, chest symptoms, history of pulmonary disease, blood pressure (BP), heart rate (HR), respiration rate (RR), body temperature (BT), peripheral oxygen saturation (SpO_2_), ETCO_2_, PaCO_2_, radiologic finding of simple chest *X*-ray and chest CT, surgical management of pneumothorax, duration of hospital stay, and mortality. Spontaneous pneumothorax that occurred without evidence of underlining lung disease was defined as PSP. Pneumothorax that occurred as a complication of underling lung disease that altered normal lung structure was defined as SSP. Underlining lung diseases were confirmed by past medical history or by findings of chest CT reviewed by an independent radiologist. They were categorized as abnormal parenchyma (including known lung disease (eq. COPD, cystic fibrosis, interstitial lung disease, and bronchiectasis), including radiologic finding of emphysema, bullous emphysema, sequelae of tuberculosis/except simple subpleural blebs or single bulla), infection, malignancy, or others (endometriosis, genetic predisposition, etc.). Both current smokers and ex-smokers were defined as smokers. An initial systolic BP (SBP) less than 90 mmHg or diastolic BP (DBP) less than 60 mmHg after ER visit was defined as low BP. An initial HR of less than 60 or more than 120 beats per minute after ER visit was defined as an abnormal heart rate. An initial RR more than 20 per minute after ER visit was defined as tachypnea. An initial BT of 37.3°C or more than 37.3°C after ER visit was defined as fever. An initial SpO_2_ of less than 90% after ER visit was defined as desaturation. The pneumothorax diagnosed for first time in life of patient was defined as the first episode. A distance of more than 2 cm between the parietal and visceral pleura at the level of the hilum in chest *X*-ray was defined as a large-size pneumothorax according to British guidelines. Percent of pneumothorax was calculated by rhea and choi method [PA projection: pneumothorax% = 4.95 + 8.8 (apical interpleural distance + interpleural distance of midpoint of upper half of lung + interpleural distance of midpoint of lower half of lung)/3, and AP projection: pneumothorax% = 9 + 10 (apical interpleural distance + interpleural distance of midpoint of upper half of lung + interpleural distance of midpoint of lower half of lung)/3] [8, 9]. The presence of pneumothorax in both lungs was defined as bilateral pneumothorax. Subsequent chemical pleurodesis or wedged resection via video-assisted thoracic surgery after closed tube thoracotomy was defined as subsequent surgical operation. Initial SpO_2_ was defined as initially measured percent of oxygen saturation by peripheral pulse oximetry after ER visit. Initial ETCO_2_ was defined as initially measured partial pressure of ETCO_2_ after ER visit. Initial PaCO_2_ was defined as partial pressure of carbon dioxide on initial arterial blood gas analysis after ER visit. Pa-ETCO_2_ gradient was calculated as the difference between initial ETCO_2_ and initial PaCO_2_ (Pa-ETCO_2_ gradient = initial ETCO_2_—initial PaCO_2_). The ETCO_2_ after air drainage was defined as the average value of ETCO_2_ measured between 45 and 75 minutes after tube thoracotomy. ETCO_2_ rise after air drainage was defined as increase of ETCO_2_ after closed tube thoracotomy (ETCO_2_ rise after air drainage = ETCO_2_ after tube thoracotomy—initial ETCO_2_).

### 2.3. Statistical Analysis

We compared study variables between PSP and SSP groups. Continuous variables are presented as median values (interquartile range, IQR) and compared with the Mann-Whitney test. Nominal data were calculated as percentages based on the frequency of occurrence and compared using Chi-squared test or Fisher's exact test, as appropriate. Multivariate logistic regression was used to correlate gas variables with SSP. The area under receiver operating characteristic (ROC) curve of ETCO_2_ for the detection of SSP was calculated. Resulting odds ratios (ORs) are presented with 95% confidence intervals (95% CIs). A two-sided *p* value of less than 0.05 was considered statistically significant.

### 2.4. Ethical Statement

This study was approved by the Institutional Review Board of Dongguk University Ilsan Hospital, Dongguk University (2020-09-025). Informed consent was waived by the IRB due to its retrospective nature.

## 3. Results

A total of 66 patients were diagnosed as spontaneous pneumothorax in ED from April 2019 to September 2020. Of these, 16 were excluded due to no ETCO_2_ monitoring (*n* = 11) or no chest CT scan (*n* = 5). Finally, 50 patients were enrolled for the analysis. They were divided into two groups: 33 (66%) in the PSP group and 17 (34%) in the SSP group.

### 3.1. General Characteristics

Patients in the SSP group were older than those in the PSP group (56 (50–70) years vs. 20 (17–29) years, *p* ≤ 0.001). Smokers were not significantly different between the two groups. However, those in the SSP group had longer smoking duration than those in the PSP group (29 (15–35) pack years vs. 5 (3–5) pack years, *p*=0.012). In the SSP group, all of the cases showed abnormal finding on chest CT; the underlying lung disease was abnormal parenchyma in 10 cases, infection in 8 cases, malignancy in 2 cases, and endometriosis in 1 case. Abnormal heart rate (5 (29.4%) vs. 1 (3.0%), *p*=0.014), desaturation (4 (23.5%) vs. 0, *p*=0.010), and bilateral pneumothorax (4 (23.5%) vs. 0, *p*=0.010) were more in the SSP group than in the PSP group. There were no significant differences in gender, symptoms (pleuritic chest pain, dyspnea), onset-to-visit interval, low BP, tachypnea, fever, first episode pneumothorax, or size of pneumothorax between the two groups. The SSP group received more tube thoracotomy than the PSP (21 (63.6%) vs. 16 (94.1%), *p*=0.038). The SSP group had a longer hospital day than the PSP group (8 (6–12) days vs. 6 (3–7) days, *p*=0.003). There were no significant differences in subsequent surgical operation or mortality between the two groups (Table 1).

### 3.2. Comparison of Respiratory Gases between PSP and SSP Groups

Initial ETCO_2_ was lower in the SSP group than in the PSP group (30 (23–33) mmHg vs. 35 (33–38) mmHg, *p*=0.002). Pa-ETCO_2_ gradient was higher in the SSP group (11.4 (5.7–18.3) mmHg vs. 3.3 (0.6–6.7) mmHg, *p*=0.001). The ETCO_2_ level rise after closed thoracotomy was higher in the SSP group than in the PSP group (6 (3–7) mmHg vs. 0 (0–3) mmHg, *p*=0.008). However, initial PaCO_2_ had no significant difference between the two groups (Table 2).

Multivariate logistic regression revealed that respiratory gas associated with SSP was initial ETCO_2_ (OR: 0.824; 95% CI: 0.697–0.974, *p*=0.023) (Table 3).

**Figure 1:**
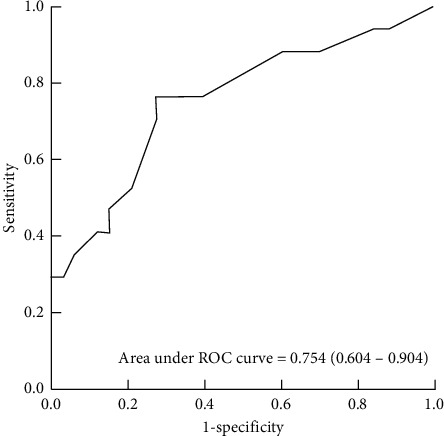
Receiver operating characteristic (ROC) curve of the initial ETCO_2_ for the detection of SSP. ETCO_2_, end-tidal carbon dioxide; SSP, secondary spontaneous pneumothorax.

Initial ETCO_2_ had an area under the ROC curve (AUC) of 0.754 (CI: 604–0.904), with lower initial ETCO_2_ values indicating SSP. The optimal cutoff for initial ETCO_2_ to detect SSP was 32 mmHg, with a sensitivity of 76.5% and a specificity of 72.7% (Figure 1).

## 4. Discussion

Lower initial ETCO_2_ was associated with SSP in this study. Initial ETCO_2_ could be an acceptable factor for discriminating between PSP and SSP. If there is a known lung disease or if the lung abnormality is evident in the chest *X*-ray conducted as a primary exam, SSP can be easily determined. A screening factor to determine SSP may not be necessary in such cases. However, because there are also many unrecognized pulmonary diseases and ambivalent findings, especially for those with collapsed lung due to pneumothorax in chest *X*-ray, it is not easy to distinguish between PSP and SSP in such cases. [10] The chest CT scan was routinely performed to evaluate underlying lung disease that may have contributed to the disease course in our institution; in our study, 8 (47.1%) cases showed these unrecognized parenchyma abnormalities in the SSP group who did not have known lung disease but showed lung abnormality in chest CT scan. Although chest CT has the advantage of being able to easily detect parenchymal changes in lung, it is difficult for chest CT to help clinician make a rapid decision because it is not a primary bedside test, and it can be limitedly performed due to the incurring cost and radiation exposure. In addition, 2001 American College of Chest Physicians [11] recommended selective use of chest CT scan in cases with recurrent pneumothorax, persistent air leak, or planning surgical intervention such as lung volume reduction surgery. On the other hand, the use of side-stream ETCO_2_ is reasonable to screen SSP because it is noninvasive, there is no radiation hazard, there is no additional time-consuming procedure for monitoring, and it can be bedside monitored in real time without interfering with necessary surgical procedures.

As shown in our study results, old age and smoking were risk factors for SSP. SSP was more often expressed by unstable features such as abnormal heart rate, hypoxia, and bilateral pneumothorax, resulting in more need for tube thoracotomy and longer hospital stays. In SSP, clinical course may worsen to respiratory failure due to an increase in V/Q mismatch caused by additional alveolar hypoventilation in the existing dysfunctional lung [12]. A few studies have revealed a change of ETCO_2_ in spontaneous pneumothorax [13]. Some case reports have shown that ETCO_2_ can be reduced in tension pneumothorax. The mechanism has been explained by a decrease in cardiac output due to a decrease in venous return [14]. In our study, there was no statistical difference in low blood pressure between the two groups. Thus, it was not enough to explain the difference in ETCO_2_ by decreased cardiac output. ETCO_2_ can change due to respiratory dynamics such as respiratory volume, in which case PaCO_2_ also changes. However, in our study, since PaCO_2_ did not show any significant difference between SSP and PSP, respiration volume could be excluded as the cause of ETCO_2_ reduction. According to Bohr equation [15], the fraction of alveolar dead space/tidal volume is equal to the fraction of Pa-ETCO_2_/PaCO_2_. Therefore, the change in Pa-ETCO_2_ gradient correlates with the change in the alveolar dead space [16]. Using this theory, some studies have revealed that the severity of pulmonary diseases with increased dead space can be clinically classified based on Pa-ETCO_2_ gradient such as acute respiratory distress syndrome [5]. In results of our study, the Pa-ETCO_2_ gradient was higher in SSP than in PSP with a normal gradient. This might be due to a V/Q mismatch in SSP. In addition, the reason for an increase in ETCO_2_ after air drainage management in SSP might be improved pulmonary dysfunction. Moran et al. [17] have performed an animal study and reported that hypoxia may occur by anatomical shunt in unilateral pneumothorax. However, the concentration of CO_2_ is unchanged because minute ventilation is maintained by controlling the respiration rate and V/Q balance is restored by autoregulation. This theory could explain why CO_2_ variable did not change in PSP in our results.

Several studies have shown that ETCO_2_ can be used to predict disease severity and prognostication in acute phase of systemic diseases such as sepsis, trauma, and cardiac arrest [18, 19]. However, our study could not reveal whether severity or prognosis of pneumothorax was related to ETCO_2_ because we did not have enough subjects. In addition, our results showed an increase of ETCO_2_ after closed thoracotomy. From these results, it could be assumed that ETCO_2_ will drop when air is refilled in the interpleural space by tube dysfunction such as blockage or tube kinking. Therefore, further study is needed to verify the effectiveness of ETCO_2_ for monitoring tube function.

This study has some limitations. First, because of its retrospective design, the time for recording the initial ETCO_2_ and the time for sampling PaCO_2_ were not exactly the same. Thus, sudden changes of variables if patients were unstable could not be reflected. Still, the time difference between sampling and recording was limited to within 30 minutes (median, 9 min; IQR, 2–20 min). Also, PaCO_2_ values were not measured in all patients. This is because the decision of whether or not to perform the arterial blood gas test was performed differently depending on the condition of each patient and the policy of each clinician. Second, 50% of patients had no record of ETCO_2_ rise after air drainage on ER because they did not need closed tube thoracotomy or they underwent closed tube thoracotomy after admission. Depending on the situation of procedure room on ER or the preference of the physician or the patient's condition, the place for performing tube thoracotomy may change. Therefore, this study might have a risk of selection bias in the analysis of ETCO_2_ rise. Third, in this study, we assumed that underlying pulmonary dysfunction might be present based on past history and radiologic finding without quantifying. However, in pneumothorax attack, performing forced expiratory maneuvers for pulmonary function test is ill-advised with unstable condition. It may produce inaccurate results with dynamic condition change in an ED setting. Finally, comprehensive factors of respiratory dynamics and hemodynamics have not been presented in our study, such as minute volume and cardiac output, which may be related to ETCO_2_. However, there were limitations because of retrospective study design, measuring some of these variables may require specific procedures, and accurate respiration volume is difficult to measure unless fixed in a laboratory setting. And the relationship between cardiac output and ETCO_2_ is logarithmic, and it is known that ETCO_2_ also changes when the cardiac output reduction exceeds the normal range enough to cause significant shock [20]. Since there was no difference in PaCO_2_ and low BP between PSP and SSP groups in our results, it could be estimated that there is no significant difference in respiratory dynamics and hemodynamics that may affect ETCO_2_ between the two groups.

## 5. Conclusions

The ETCO_2_ monitoring in spontaneous pneumothorax may be an acceptable practice for discriminating between PSP and SSP. Lower initial ETCO_2_ is associated with SSP. Its optimal cutoff value is 32 mmHg (AUC: 0.754, sensitivity: 76.5%, specificity: 72.7%). Further research addressing the usefulness of ETCO_2_ as a severity and prognostic indicator for spontaneous pneumothorax is needed.

## Figures and Tables

**Table 1:** Comparison of general characteristics between PSP and SSP groups.

	Total (*n* = 50)	PSP (*n* = 33)	SSP (*n* = 17)	*p* value
Age (yr)	29 (19–51)^*∗*^	20 (17–29)^*∗*^	56 (50–70)^*∗*^	<0.001
Male gender, no. (%)	37 (74.0)	27 (81.8)	10 (58.8)	0.099
Smoker, no. (%)	16 (32.0)	10 (30.3)	6 (35.3)	0.757
Pack years among smokers	5 (3–22)^*∗*^	5 (3–5)^*∗*^	29 (15–35)^*∗*^	0.012

*Symptoms*				
Pleuritic pain, no. (%)	17 (34.0)	14 (42.4)	3 (17.6)	0.117
Dyspnea, no. (%)	22 (44.0)	17 (51.5)	11 (64.7)	0.548
Onset-to-visit interval (hr)	10 (4–24)^*∗*^	8 (4–24)^*∗*^	12 (2–28)^*∗*^	0.734

*Underlying lung disease in SSP*				
Abnormal parenchyma, no. (%)			10 (58.8)	
Infection, no. (%)			8 (47.1)	
Malignancy, no. (%)			2 (11.8)	
Pulmonary endometriosis, no. (%)			1 (5.9)	
Initial SBP <90 mmHg or initial DBP <60 mmHg	9 (18)	5 (15.2)	4 (23.5)	0.467
Initial HR ≥120 or <60 rate/min	6 (12.0)	1 (3.0)	5 (29.4)	0.014
Initial RR > 20 rate/min	6(12.0)	2 (6.1)	4 (23.5)	0.161
Initial BT ≥37.3°C	1 (2.0)	0	1 (5.9)	0.340
Initial room-air SpO_2_ <90%	4 (8.0)	0	4 (23.5)	0.010
First episode PNX, no. (%)	14 (28.0)	12 (36.4)	2 (11.8)	0.099
Bilateral PNX, no. (%)	4 (8.0)	0	4 (23.5)	0.010

*Size of PNX*				
Percentage of PNX	34.0 (15.0–53.3)^*∗*^	29.4 (15.0–52.6)^*∗*^	35.1 (23.0–72.1)^*∗*^	0.529
Large-size PNX, no. (%)	20 (40.0)	13 (39.4)	7 (41.2)	1.000
Closed tube thoracotomy, no. (%)	37 (74.0)	21 (63.6)	16 (94.1)	0.038
Subsequent surgical operation, no. (%)	22 (44.0)	17 (51.5)	5 (29.4)	0.229
Hospital days	6 (3–8)^*∗*^	6 (3–7)^*∗*^	8 (6–12)^*∗*^	0.003
Mortality, no. (%)	1 (2.0)	0	1 (5.9)	0.340

^*∗*^Median (interquartile range); PSP, primary spontaneous pneumothorax; SSP, secondary spontaneous pneumothorax; SBP, systolic blood pressure; DBP, diastolic blood pressure; HR, heart rate; RR, respiration rate; BT, body temperature; SpO_2_, peripheral oxygen saturation; PNX, pneumothorax.

**Table 2:** Comparison of CO_2_ variables between PSP and SSP groups.

	Total	*N*	PSP	*N*	SSP	*N*	*p* value
Initial ETCO_2_ (mmHg)	34 (30–38)^*∗*^	50	35 (33–38)^*∗*^	33	30 (23–33)^*∗*^	17	0.002
Initial PaCO_2_ (mmHg)	38.6 (36.7–41.1)^*∗*^	47	38.5 (36.7–41.0)^*∗*^	31	39.6 (32.9–44.6)^*∗*^	16	0.685
Pa-ETCO_2_ gradient (mmHg)	5.6 (1.0–10.4)^*∗*^	47	3.3 (0.6–6.7)^*∗*^	31	11.4 (5.7–18.3)^*∗*^	16	0.001
ETCO_2_ rise after air drainage (mmHg)	2 (0–6)^*∗*^	25	0 (0–3)^*∗*^	16	6 (3–7)^*∗*^	9	0.008

^*∗*^Median (interquartile range); PSP, primary spontaneous pneumothorax; SSP, secondary spontaneous pneumothorax.

**Table 3:** Multivariate analysis of respiratory gases associated with SSP.

Respiratory gases	Odds ratio	95% CI	*p* value
Room-air SpO_2_ (%)	0.837	0.638–1.098	0.200
Initial PaCO_2_ (mmHg)	1.094	0.945–1.267	0.227
Initial ETCO_2_ (mmHg)	0.824	0.697–0.974	0.023

SSP, secondary spontaneous pneumothorax; CI, confidence interval.

## Data Availability

All datasets used and/or analyzed in the current study are available from the corresponding author upon reasonable request.
